# Setting meaningful goals in rehabilitation: A qualitative study on the experiences of clients and clinicians in working with a practical tool

**DOI:** 10.1177/02692155211046463

**Published:** 2021-11-03

**Authors:** Elsbeth Littooij, Suzan Doodeman, Jasmijn Holla, Maaike Ouwerkerk, Lenneke Post, Ton Satink, Anne Marie ter Steeg, Judith Vloothuis, Joost Dekker, Vincent de Groot

**Affiliations:** 1 Amsterdam Rehabilitation Research Center, Reade, the Netherlands; 2 Department of Rehabilitation Medicine, Amsterdam University Medical Centers, the Netherlands; 3 Nieuw Unicum, the Netherlands; 4 Department of Spiritual Care, Amsterdam University Medical Centers, the Netherlands; 5 Faculty of Religion and Theology, VU University, the Netherlands; 6 Department of Occupational Therapy & Research Group Neurorehabilitation, HAN University of Applied Sciences, the Netherlands

**Keywords:** Goal-setting, rehabilitation, meaning, motivation, behavior change

## Abstract

**Objective:**

To evaluate the experience of clients and clinicians in working with a tool to help set goals that are personally meaningful to rehabilitation clients.

**Design:**

We have applied the tool in the outpatient rehabilitation setting. Clients’ and clinicians’ experiences in working with the tool were evaluated in individual, semi-structured interviews and focus group interviews, respectively. Thematic analysis was used to analyze the data.

**Setting:**

A university medical center and a rehabilitation center.

**Subjects:**

Clients with a first-time stroke (*n* = 8) or multiple sclerosis (*n* = 10), and clinicians (*n* = 38).

**Intervention:**

The tool to help set meaningful goals consisted of a session (i) to explore the client's fundamental beliefs, goals and attitudes and (ii) to identify a meaningful overall rehabilitation goal. The results of that session were used by the multidisciplinary rehabilitation team (iii) to help the client to set specific rehabilitation goals that served to achieve the meaningful overall rehabilitation goal.

**Results:**

Both clients and clinicians reported that the tool helped to set a meaningful overall rehabilitation goal and specific goals that became meaningful as they served to achieve the overall goal. This contributed to clients’ intrinsic rehabilitation motivation. In some clients, the meaningfulness of the rehabilitation goals facilitated the process of behavior change. Both clients and clinicians made suggestions on how the tool could be further improved.

**Conclusion:**

In the opinion of both clients and clinicians, the tool does indeed result in goal setting that is personally meaningful. Further development, implementation and evaluation of the tool is warranted.

## Introduction

Goal-setting is a core practice within rehabilitation. Goal setting is thought to motivate the client for rehabilitation and to increase behavior change.^1^ Goal-setting is also thought to have an impact on the rehabilitation process: it is intended to ensure that individual team members work towards the same goals, and that appropriate treatment is provided.^1^ Despite it being a core practice, both clients and clinicians have reported difficulty in setting goals that are personally meaningful to clients.^2–4^ Clients are unlikely to be strongly motivated for rehabilitation if goals are not personally meaningful. This is expected to lead to low adherence and little behavior change.^1,3,5^ Therefore, there is a need for a tool to help set goals that rehabilitation clients experience as personally meaningful.

**Figure 1.**
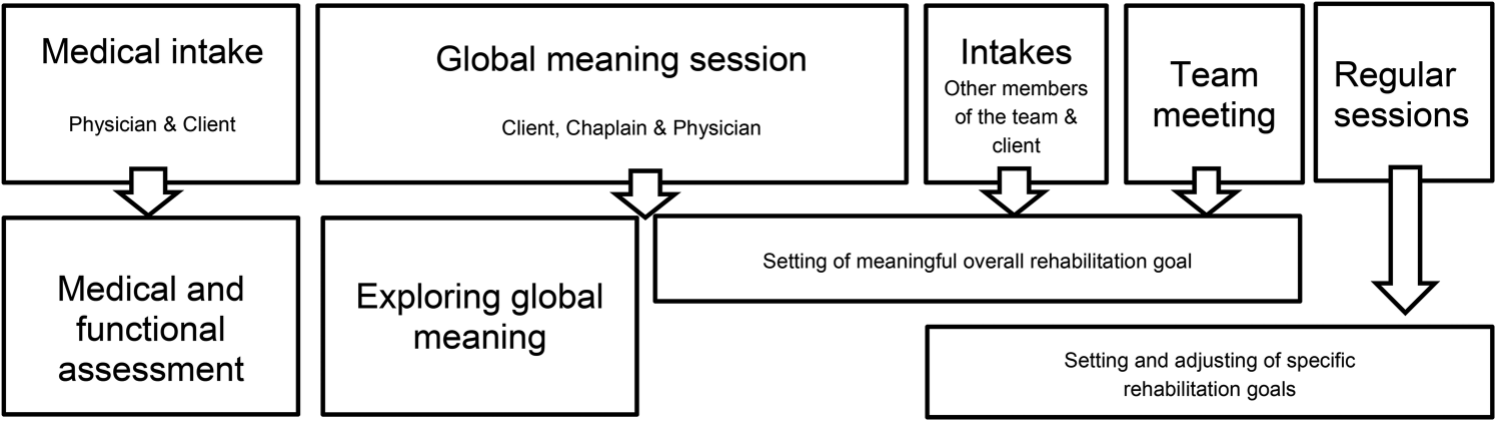
A tool to set meaningful goals in rehabilitation (reproduced from Clinical Rehabilitation^6^).

We have previously reported on the development of such a tool.^6^ The tool's central tenet is that the client's fundamental beliefs, goals and attitudes need to be explored before setting any rehabilitation goal. The exploration of these fundamental beliefs, goals and attitudes (or ‘global meaning’) enables the client to set a meaningful overall rehabilitation goal (e.g. ‘*to be and remain important to others, and to pursue my creative hobbies, for which I need to be more stable physically*’). Once the meaningful overall rehabilitation goal has been identified, the rehabilitation team and the client set more specific rehabilitation goals that serve to achieve the overall goal (e.g. ‘*within five weeks, it is clear which ankle-foot orthosis is most beneficial for me with the least drawbacks*’). During the rehabilitation trajectory, the specific goals can be adjusted or revised, depending on the course of the rehabilitation process. Our approach has been to involve a chaplain to support the client in exploring their fundamental beliefs, goals and attitudes and setting the overall meaningful rehabilitation goal, although is a possibility to train other professionals to do this. The tool to help set meaningful goals in rehabilitation is illustrated in Figure 1.

After its development, evaluating the tool is the next logical step. The aim of the present study was a formative evaluation of the experience of clients and clinicians (rehabilitation physicians, chaplains and other members of the rehabilitation team) in working with the tool. The evaluation focused on meaningfulness of rehabilitation goals, clients’ motivation and related behavior change, as well as the rehabilitation work process. We aimed to conduct the study among clients with a first-time stroke (a sudden onset condition), multiple sclerosis (a progressive condition) and cerebral palsy (a lifelong condition), to get an impression about the use of the tool in conditions with different time course. The study was conducted in the outpatient setting.

## Methods

In a qualitative study, we evaluated clients’ and clinicians’ experiences in using the tool in the outpatient rehabilitation clinics of the Amsterdam University Medical Centers and Reade, Center for Rehabilitation and Rheumatology in Amsterdam. The Medical Ethics Committee of Amsterdam University Medical Centers declared that the Research Involving Human Subjects Act did not apply and that official approval of the study by the committee was not required (reference number 2018.013). Clients were recruited between October 2018 and August 2019.

At both clinics, rehabilitation physicians were invited to participate in the study, based on oral information and a brief presentation. Rehabilitation physicians invited clients to participate in the study. Three chaplains were involved who helped to explore global meaning and to set the overall meaningful rehabilitation goal (see next paragraph). We aimed to include adult clients with a first-time stroke (*n* = 10), multiple sclerosis (*n* = 5), or cerebral palsy (*n* = 5). We expected to get enough information with these number of clients, without exceeding our capacity to apply and evaluate the tool. Clients with severe communication problems or psychiatric problems were excluded. Physicians applied purposive sampling to include both men and women, and both younger and older clients.

If the client agreed to participate, the researchers sent an information letter and a consent form. In the letter, the study goal and procedures were explained. After the client had signed the consent form, the session on global meaning was planned, at the start of outpatient rehabilitation after the medical intake (see Figure 1). In that session, the client, chaplain and rehabilitation physician (i) explored the client's global meaning (the client's relationships, core values, worldview, identity and inner posture^6^), and (ii) together they identified the meaningful overall rehabilitation goal. Additional support by the chaplain was offered in case the session on global meaning raised questions or feelings the client wanted to elaborate on. The results of that session were briefly summarized; after a check with the patient, the summary was included in the client's file and communicated to the other members of the rehabilitation team. The other members of the rehabilitation team and the client (iii) set specific rehabilitation goals that served to achieve the meaningful overall rehabilitation goal. The specific goals were recorded in the client's file. During the rehabilitation trajectory, the specific goals could be adjusted or revised, depending on the course of the rehabilitation process. For further details on the process of setting meaningful goals, we refer to our previous publication.^6^

The experiences of clients were evaluated in individual, semi-structured interviews, in the final phase of their rehabilitation process (3–6 months after the start of outpatient rehabilitation). Clinicians’ experiences were assessed in multidisciplinary focus groups, in the final phase of data collection. The individual interviews and focus group interviews were loosely structured using a topic list. The topic list concerned: (i) the conversation about global meaning; (ii) the incorporation of global meaning into the process of goal setting; (iii) re-setting of specific goals during the process of rehabilitation, taking into account the overall goal; and (iv) the impact on the process of rehabilitation, in particular the impact on the client's motivation for rehabilitation (Supplemental file 1). Audio-recording and field notes were made. Two chaplain-researchers (EL and SD) conducted the interviews and focus groups. They had a background in qualitative research, one of them had a PhD, and both were of the female gender. As they were also involved in exploring clients’ global meaning and setting the overall rehabilitation goal, each chaplain-researcher interviewed the other chaplain-researcher's clients, in order to encourage clients to speak freely about their experiences with someone who was not involved in the session on global meaning. The focus groups were guided by the two researchers together. Occasionally, the researchers attended the team meeting regarding the included clients and made field notes about goal setting and treatment planning.

Transcripts from the interviews and focus groups were analyzed by the two chaplain-researchers, using thematic analysis.^7^ They read all transcripts to familiarize themselves with the data. The researchers coded the interviews (open coding), using the qualitative data analysis software Atlas.ti. They collated codes into potential themes, gathering all codes relevant to each potential theme. They reviewed, defined and named the themes. The field notes about goal setting and treatment planning obtained during the team meetings were added to the data of clinicians. The interviews with clients and focus groups with clinicians were analyzed separately, in order to ensure an independent analysis.

To ensure the trustworthiness of the analysis, several strategies were applied.^8^ First, peer review was applied to enhance credibility. The two researchers compared and discussed the individual steps in data collection and analysis. Throughout the process, they discussed their opinions and findings, until mutual agreement was reached. Besides that, they regularly discussed their findings within the whole research group. Second, regarding reflexivity: the researchers documented decisions regarding the analysis and the development of themes. The decisions were based on a critical discussion with other members of the team. Third, a member check was applied. A short summary of the interview and the initial analysis was sent to clients and clinicians, who provided feedback regarding the summary and initial analysis. Fourth, in the initial phase of the analysis an independent researcher (MO) analyzed two interviews with clients and one focus group. The result of these analyses was discussed and compared to the analyses of the two main researchers.

## Results

### Participants

In total 38 clinicians participated in the study: eight rehabilitation physicians (5 and 3 in centers A and B, respectively), three chaplains (1 and 2), and 27 other members of the rehabilitation team (20 and 7). The category of physicians also comprised residents and a physician assistant. The category of other members of the team comprised physical therapists (7 and 1 in centers A and B, respectively), occupational therapists (5 and 2), speech therapists (2 and 0), sports therapists (1 and 0), psychologists (3 and 3) and social workers (2 and 1). In total 18 clients participated in the study (see Table 1; we did not monitor whether patients were approached but then refused to participate). Clients had a diagnosis of first-time stroke (*n* = 8) or multiple sclerosis (*n* = 10). Clients with cerebral palsy were not recruited (as none had a health problem requiring multidisciplinary rehabilitation). Three clients initially agreed to participate, but withdrew from the study (one without a reason given, one because (s)he thought study participation ‘would be too much’, and one because (s)he was referred to another hospital).

**Table 1.** Clients’ characteristics.

		Total	Center A	Center B
Number of clients initially included		21	13	8
Withdrawal		3	3	0
Gender	Male	7	7	0
	Female	11	3	8
Age (years)	Mean	46	48	44
	Range	23–67	23–67	24–58
Diagnosis	First-time stroke	8	8	0
	Multiple sclerosis	10	2	8

All clients participated in the interviews to report their experiences. In two cases, a family member was present during the interview. All clinicians were invited to participate in the focus groups; due to scheduling issues, not all clinicians could participate: 13 clinicians participated in the focus groups and 14 clinicians participated in an individual interview (in total 27 out of 38 clinicians). Clinicians were asked to report the experiences of other clinicians with the same disciplinary background as well (only sports therapists were not represented). The interviews and focus groups were conducted at the clinic (except for two interviews that were held at home). The interviews ran for about 45 min, the focus groups for 1 h.

### Themes

The following themes emerged in the interviews and focus groups: overall evaluation, impact on motivation, impact on behavior change, stability of global meaning and goals, existential distress, and process of setting meaningful goals. Box 1 provides an overview of the themes, with featured quotes. Supplemental file 2 provides an overview of the development of the themes.

**Box 1.** Overview of the themes in the evaluation of the tool, with featured quotes.

Themes	Featured quotes – clients	Featured quotes – clinicians
Overall evaluation	Clients’ experiences ranged from very positive to neutral. ‘*It was about me, not only about the multiple sclerosis, it was really about me.’* (M1) ‘*The questions were to the point and it made me aware of what I myself hoped and expected of my rehabilitation. Every patient should have a conversation like this, this is what rehabilitation is all about.’* (M10) ‘*It was okay but had no impact on my rehabilitation.’ (M7)*	Clinicians were unanimous in their belief that meaning is important in rehabilitation. ‘*I was able to attune more deliberately to my patient's needs and goals.’* (physician) ‘*I think it is a form of investment in the collaboration with the patient when we explicitly address these things [global meaning]. We show them that they matter, that we really want to know who they are and what is important to them.’* (social worker) ‘*I noticed that the whole team started thinking on that level <of global goals> again and not just focus on their own discipline.’* (physician assistant) Clinicians varied in their appreciation of the tool. ‘*Engaging in this conversation created more awareness and prompted me to ask just that one extra question.’* (physician) ‘*The meaningful overall goals I read in the summary were similar to the ones in my own assessment’.* (occupational therapist)
		
Impact on motivation	Clients frequently mentioned the connection between global meaning and motivation. Referring to values or relationships they talked about in the session on global meaning, clients regularly told themselves: ‘*This is what I want to accomplish and this is why’*. (S4, M4)	Clinicians noticed the impact of the session on global meaning on motivation. ‘*With one patient, we agreed that she would go to the gym with her sister, instead of doing her exercises here. She could not motivate herself to come here, because she didn't want to be a patient. Going to the gym with her sister worked for her.’* (physical therapist)
Impact on behaviour change	A client continued to be active in her sports, but in an adapted way, and based on another part of her global meaning. At first, her activity in sports was based on the value of self-enhancement and the identity of a winner: ‘*I am someone who wants to win’. After the session and her rehabilitation, it was based more on the value of enjoying the activity and on relationships, being part of a group: ‘if I can participate in small parts, and enjoy it, then it's okay’*. (M1)	Clinicians saw global meaning as an important source of intrinsic motivation for behaviour change. ‘*I think that global meaning can help clarify a person's intrinsic reason for change. It is important to find and use that reason’.* (social worker) *‘A patient's being is what should be the source of their motivation. I would never set a goal which does not involve a person's global meaning, because it gives you very little chance of maintaining the changed behavior.’* (physical therapist)
Stability of global meaning and goals	Clients experienced global meaning and meaningful overall goals as stable, and adapted specific goals using meaningful overall goal as a reference. ‘*Those three goals, whether I can really reach them remains the question, but those are the things that make life meaningful to me. That remains the same.’* (S4) ‘*I expressed myself to the world, first by making movies, now by making blogs. At first I could still type my own texts, now I need to dictate and some days my voice isn't even good enough for that. But that is important to me: expressing myself to the world.’* (M6) ‘*I am pleased with myself because this week I went running and I enjoyed it. I used to be pleased because I was faster than other people. I am not faster anymore. But I still am pleased with myself, and that is what is important to me.’* (S2)	Clinicians used the stable overall goals to provide direction for the specific goals. ‘*Goals do change. Every six weeks we set new rehabilitation goals, because someone is in another phase. But our overall rehabilitation treatment, that we agree “we are going in that direction,” that did not change.’* (physician assistant)
Existential distress	One client found the summary of her global meaning so confronting, that she did not want her partner or anybody else to read it. ‘*I thought no, this is not who I am! I thought: this is out of proportion, I am not in as bad a state as these words suggest. So I never reacted to the question whether this was okay to put into my file. The description was correct, though. I said those things. But I don't want anyone to read them and perhaps use against me.’* (M8)	Diminished physical and mental capacities led to confusion and distress in a client, raising questions such as: ‘*Who am I, what is my value in life, now that I cannot contribute the way I did.’* (physician)
Process of setting meaningful goals	The session on global meaning came at the right time for most clients. *‘It helped me prioritize and keep agency over my rehabilitation process.’* (S4) Some clients stated that the global meaning session came too early: *‘Doing this interview created a dilemma. Isn't it too much for me? Should I do it because I promised? I want to make things easy for myself. I need all my strength to do my therapies. I still don't know if it was the right decision to come.’* (M2)	Therapists stated that the tool did influence the way in which they approached their clients. ‘*For example, the patient that told us that he needed to be pushed: I wouldn't have known. And it worked for him.’* (physician assistant) ‘*I had the idea that it was sort of the same as what I had already recorded. I did not gain new answers or insights.’* (occupational therapist) ‘*That patient that said “I prioritize differently now”, I asked her “how does that relate to your global meaning”, and after a moment of looking at me with glassy eyes (laughs) she could answer that really well. So, I use it actively.’* (physical therapist)
		

The same themes were identified for clients and clinicians. Clinicians made more comments on the process of setting meaningful goals than clients. Clients with stroke and with multiple sclerosis made similar comments for the most part; clinicians also made similar comments about these diagnostic groups. The only exception was that some clients with multiple sclerosis stated that the global meaning session came too early (see below), while clients with stroke did not mention this as an issue.

### Clients

#### Overall evaluation

Although some clients were more eloquent than others in putting their thoughts and beliefs into words, in all cases the global meaning session resulted in a summary of the client's global meaning and overall rehabilitation goals that clients recognized themselves in. Reflecting on the global meaning session and the impact on rehabilitation, clients’ experiences ranged from ‘*it was okay but had no impact on my rehabilitation*’ to ‘*every patient should have a conversation like this, this is what rehabilitation is all about*’.

All clients appreciated the time and attention given to them as a person, rather than just rehabilitation issues. This was expressed as: ‘*It was about me, not only about the multiple sclerosis, it was really about me*’, ‘*it is good to give people food for thought, with a focus on meaning, which is different from what you get from the psychologist*’, ‘*I felt respected, seen, and understood*’, and ‘*it was no question-and-answer, but more like a real conversation*’. One client expressed her admiration that the chaplain was able to ‘*put into motion such deep thoughts in such a short time*’. Two clients stated that the global meaning session was confronting for them, but that in hindsight it was helpful and enriching. One of them even stated that ‘*you should do this with every patient, even if they don't want it, or are not able to formulate it very well. (…) everybody needs this*’.

Several clients emphasized the importance of the global meaning session in their rehabilitation. ‘*The questions were to the point and it made me aware of what I myself hoped and expected of my rehabilitation*’; ‘*I use the summary as a thermometer, I can see how my condition was when I answered these questions and where I am now*’; and ‘*it helps to structure reflection*’. On the other hand, one client stated that her goals had no connection to her global meaning, and she did not think that the global meaning session or the summary would be useful to her in the future.

#### Impact on motivation

Clients frequently mentioned the connection between motivation and global meaning; several clients explicitly referred to the global meaning session, claiming that the session had made a difference for them. During rehabilitation, they regularly told themselves ‘*this is what I want to accomplish and this is why*’, referring to values or relationships they talked about in the session on global meaning. One client used her meaningful overall goals to choose what exercises to give priority. When asked in the global meaning session what would keep him motivated, another client said that he needed to be pushed. This was part of his inner posture. Reflecting on his rehabilitation process he claimed that his therapists had taken notice of this need and had helped him to stay motivated.

#### Impact on behavior change

Global meaning and the global meaning session played a varying role in changing clients’ behavior. Some used the global meaning session to reflect on their goals, but did not change their behavior. For others, behavior change was driven by their global meaning. These clients explicitly mentioned the global meaning session as important in their process of behavior change. One client, for example, continued to be active in her sports, but in an adapted way, and based on another part of her global meaning. At first, her activity in sports was based on the value of self-enhancement and the identity of a winner: ‘*I am someone who wants to win*’. After the session and her rehabilitation, it was based more on the value of enjoying the activity and on relationships, being part of a group: ‘*if I can participate in small parts, and enjoy it, then it's okay*’.

Several clients changed the way in which they looked at themselves and what they expected of themselves, now that their possibilities had changed. One of them stated that the global meaning session had helped her in the process of saying goodbye to her old self. She said it was good to identify what is important to her, and that it helped her to regain trust: ‘*Thinking about your life goal, knowing that your children are important to you, and your parents … it gives acknowledgement, trust, you know what you go through this trouble for*’. This helped her to make decisions relating to work and her role in the family.

#### Stability of global meaning and goals

Overall, clients experienced global meaning and meaningful overall goals as stable: ‘*Those three goals, whether I can really reach them remains the question, but those are the things that make life meaningful to me. That remains the same*’. In some clients, however, the changed possibilities as a result of their medical condition led to the question whether their identity (which is part of global meaning) had changed as well.

Specific rehabilitation goals were regularly adapted during the rehabilitation process using the meaningful overall goal as a reference. For example, the meaningful overall goal of self-respect did not change, but the focus changed: not so much focusing on self-respect based on physical achievements, but on being able to handle the new situation, or being empathetic to themselves or to others. Another example: the meaningful overall goal of being active in sports did not change, but clients choose to engage in other, less physically demanding sports.

#### Existential distress

In a few clients, we found existential distress related to global meaning. One client, for example, discovered that global meaning as she would have formulated it earlier was no longer applicable. Her global meaning had not changed (yet), but the confrontation with the consequences of her medical condition led to existential questions regarding her identity and worldview. Another client found the summary of her global meaning so confronting, that she did not want her partner or anybody else to read it. It revealed a discrepancy within her global meaning, between her negative worldview and her inner posture as ‘*miss positivo*’. Several other clients also mentioned that reading the summary was confronting. All this did not lead to severe problems in the rehabilitation process. In one case, it was an indication to involve a chaplain in the rehabilitation.

#### Process of setting meaningful goals

With regard to timing, the global meaning session came at the right time for most clients (i.e. at the start of outpatient rehabilitation after the medical intake): ‘*it helped me prioritize and keep agency over my rehabilitation process*’. Some clients stated that the global meaning session came too early: they were not prepared to reflect on such personal issues while still in shock about their diagnosis, or when still fully focused on regaining physical strength. This applied in particular to clients with multiple sclerosis.

With regard to clinicians involved, one client appreciated that she already knew the physician involved in the global meaning session. Others stated that they did not have a preference for a specific person to have this conversation with.

With regard to specific goals, several clients stated that their specific rehabilitation goals would not have been different, but that the connection to their global meaning was important, as this helped to stay motivated: ‘*it was not particularly fun, they were no easy questions, but it was really helpful, because now I knew what I wanted to achieve*’. Some clients appreciated when therapists referred to their global meaning and meaningful overall goals during therapy and in setting specific rehabilitation goals. Clients would have liked to have the summary of their global meaning with them during their rehabilitation process, not only in their file, but in their own personal papers.

### Clinicians

#### Overall evaluation

Clinicians were unanimous in their belief that meaning is important, if not key, in rehabilitation. ‘*That is what we do. I cannot imagine not addressing meaning in my training and support of people*’ (social worker). Clinicians appreciated the information given in the description of the client's global meaning. ‘*I think it is a form of investment in the collaboration with the patient when we explicitly address these things [global meaning]. We show them that they matter, that we really want to know who they are and what is important to them*’ (social worker). A psychologist became aware of the centrality of clients’ meaning: ‘*One patient from an Asian country was ashamed of his disability. We repeatedly said: you don't need to be, but now I realize that it is part of his culture, of his identity. My opinion doesn't matter, this is who he is, and this is what we have to deal with*’.

However, clinicians varied in their appreciation of the tool. Some regarded global meaning as being informative of setting rehabilitation goals, but they regarded it not necessary to set the meaningful overall goal in the global meaning session. They thought that goal setting could and should take place in team meetings. Particularly in Center A, team members felt that they already had integrated meaning in their work with clients, and questioned whether the tool improved their existing practice. One occupational therapist commented: ‘*The meaningful overall goals I read in the summary were similar to the ones in my own assessment*’. On the other hand, a physical therapist working in Center B said that due to the summary, he could start his own specific treatment much faster.

In particular, physicians were enthusiastic about working with the tool: ‘*Engaging in this conversation created more awareness and prompted me to ask just that one extra question*’, and ‘*I was able to attune more deliberately to my patient's needs and goals*’. Physicians recognized that without the tool the information on meaning may have been known in the team, but was not shared with other team members on a regular basis. With the tool, the information on global meaning and meaningful overall rehabilitation goals was explicitly and coherently recorded in the client's file. This gave direction to the treatment plan and enabled physicians to monitor whether clients’ specific goals were still in line with their values. A physician in Center B expressed the hope that the tool would change the way a rehabilitation team works: not so much focusing on the expertise of each professional, but more on what is truly important to the client. Other clinicians stated that the tool indeed helped to regard meaning as a team-responsibility, and not the domain of one specific discipline: ‘*I noticed that the whole team started thinking on that level <of global goals> again and not just focus on their own discipline*’ (physician assistant).

#### Impact on motivation

Clinicians noticed in some clients an increased motivation to take agency over their rehabilitation. For example, one client decided to put one of her specific goals ‘*on hold*’ because she realized that it was less important to her at that moment. Another client could not motivate herself to do the prescribed exercises. Her physical therapist read in the summary of global meaning and overall rehabilitation goal that she desired to be more independent, and to feel less like a patient. Together they decided that she would go to the gym with her sister, instead of exercising with the physical therapist. This motivated her to regularly exercise again.

#### Impact on behavior change

Clinicians stated that global meaning is an important source of intrinsic motivation for clients to change their behavior. ‘*I think that global meaning can help clarify a person's intrinsic reason for change. It is important to find and use that reason*’ (social worker). A physical therapist said: ‘*A patient's being is what should be the source of their motivation. I would never set a goal which does not involve a person's global meaning, because it gives you very little chance of maintaining the changed behavior*’.

#### Stability of global meaning and goals

Clinicians saw changes in clients’ goals, but only in specific rehabilitation goals, not in meaningful overall goals. They mentioned that specific goals do change over time, and often become more clear. Global meaning, however, seemed to remain stable. The exception was when a discrepancy between global meaning and life experiences resulted in existential distress.

#### Existential distress

Physicians mentioned that some clients suffered from existential problems and spiritual distress, which became clear during the global meaning session. For example in one client, her identity and worldview were challenged by her diminished physical and mental capacities. This led to confusion and distress, raising questions, such as ‘*who am I, what is my value in life, now that I cannot contribute the way I did*’. Chaplains saw the global meaning session as a way to detect existential distress, leading to a referral to a psychologist or a chaplain.

#### Process of setting meaningful goals

With regard to the added value of the tool, in center A reactions were mostly neutral, apart from the physicians who were positive about the tool. One occupational therapist said ‘*I had the idea that it was sort of the same as what I had already recorded. I did not gain new answers or insights’*. In center B, the larger part of the team was positive about the tool and how it contributed to their way of providing treatment. Therapists stated that the tool did influence the way in which they approached their clients: ‘*For example, the patient that told us that he needed to be pushed: I wouldn't have known. And it worked for him*’ (physician assistant). One physician said that the global meaning session added something to their assessment: ‘*We already set goals, and we inquire about relationships, but identity, values, inner posture are extra*’. Other physicians also said they added some of the questions from the tool into their own medical assessment, in order to deepen their understanding of the client as a person. This fostered the way in which they could attune to the client.

With regard to the timing of the global meaning session, some clinicians emphasized that it should be one of the first things to explore with a client, while others advocated for the right of the client to refuse to ‘*go that deep*’. ‘*Some patients are not open to questions like that in the beginning of their rehabilitation. In that case, we should just start with the treatment and offer a global meaning session in a later phase*’ (occupational therapist).

With regard to clinicians involved, some physicians wanted to be involved themselves, because it enhanced their knowledge of and relationship with clients. Other physicians said that in the future it would be enough to read the global meaning summary in the client's file. It was commonly believed that expertise with regard to meaning in rehabilitation should be leading in deciding who is involved, rather than being of a certain discipline.

As to for whom the global meaning session would be beneficial, there was no consensus. Some physicians said it was impossible to know in advance who would benefit or not, and recommended a global meaning session for every client. Other physicians thought that they could tell in advance whether the session would have added value: they preferred to offer a global meaning session to some clients in a later stage of their rehabilitation.

## Discussion

This qualitative study shows that clients as well as clinicians felt that the tool helps to set meaningful goals in rehabilitation. They indicated that the exploration of global meaning facilitated the setting of a meaningful overall rehabilitation goal: as the summary of the client's global meaning and the overall rehabilitation goal were explicitly recorded in the client's file at the start of the rehabilitation, this information could be used to set specific goals. Specific goals were regularly adapted using the overall goal as a point of reference. Clients indicated that they were intrinsically motivated to work on specific goals, because they were aware of the overall goal: specific goals (‘SMART goals’) became meaningful because the specific goals served to achieve the overall goal. Some clients felt that the session on global meaning contributed to changing their behavior.

Overall, clients’ evaluation of the tool was positive: some clients were (very) positive, others were neutral; no client had a negative evaluation. The variation in clients’ evaluation could mean that for certain clients this tool has no added value, or that the tool should be used in a later stage of rehabilitation. Further exploration of this issue is required, as the present study did not provide any clues in this respect. Besides, further development and improvement of the tool is needed. This relates in particular to behavior change. Many clients indicated that the session on global meaning and the overall rehabilitation goal contributed to rehabilitation motivation; however, only some clients indicated that the increased motivation translated into behavior change. The application of behavior change theory and techniques could result in a better translation of motivation into actual behavior change. For example, the Health Action Process Approach^9,10^ provides theoretical guidance on how to translate intentions (motivation) into action. In addition, practical behavior change techniques are available that can be used to help motivated clients actually change their behavior.^11^

Clinicians also varied in their evaluation, ranging from neutral to (very) positive. Physicians were generally positive about the tool, in both centers. Other members of the team were more mixed in their evaluation. Some team members felt that they already had integrated meaning in their work, and questioned whether the tool improved their existing practice, particularly in center A. Indeed, occupational therapists, social workers and other disciplines have embraced client-centered practice, which entails the exploration of clients’ values and priorities.^12,13^ The advantage of working with our tool is that global meaning is explicitly addressed; that global meaning is addressed in a comprehensive way (focusing on fundamental beliefs, goals and attitudes^14–17^); and that the summary of the client's global meaning and the overall rehabilitation goal is explicitly recorded in the client's file at the start of the rehabilitation process. The present study also indicated that the tool helped to regard meaning as a team-responsibility, and not the domain of one specific discipline. In the future implementation of the tool, we recommend involving all team members at an early stage, to remove implementation barriers and to increase support for working with the tool. The application of a knowledge translation framework^18^ and practical techniques such as co-design^19^ may help to change the behavior of rehabilitation clinicians.

Some physicians expressed a desire to participate in the global meaning session themselves, as this contributed to their knowledge of and relationship with clients. According to other physicians, another member of the team could participate in the session (e.g. an occupational therapist or social worker). Similarly, the need for a chaplain to be involved could be reconsidered. Another member of the team could be trained to explore global meaning (e.g. a psychologist, occupational therapist or social worker). For the session to be successful, the session must include knowledge of the client's medical condition and care pathway; expertise in exploring global meaning is also required. This would argue for the coordinating physician or physician assistant, and the chaplain or someone with similar expertise to be present in the session on global meaning.

Overall, clients and clinicians mentioned the same themes in their evaluation of the meaningful goal-setting tool. Clinicians made more comments on the process of setting meaningful goals than clients. Possibly, clinicians were more concerned about the process of rehabilitation. There were no notable differences in the evaluation between diagnostic groups (stroke and multiple sclerosis), not among clients, nor among clinicians. The only exception concerns the timing of the session on global meaning and the overall rehabilitation goal. For some clients with multiple sclerosis, this came too early: some clients with multiple sclerosis were referred for rehabilitation soon after receiving the diagnosis; they were still in the process of accepting the implications of their diagnosis.

In rehabilitation, the role of clients’ values and perspectives has been increasing recognized over the last decades. Examples of approaches taking clients’ values and perspectives into account include patient-centered care,^12,13^ acceptance and commitment therapy^20^ and spirituality and religion-based interventions.^21–23^ We believe that our approach is more broad and encompassing than these approaches. Our tool is based on the exploration of global meaning, that is, the client's fundamental beliefs, goals and attitudes.^14–17^ The session on global meaning concerns the client's relationships, core values, worldview (including spirituality and religion), identity and inner posture.^6^ We believe that this broad approach contributed substantially to the identification of a meaningful overall rehabilitation goal, and thereby the success of our approach.

Several methodological issues need to be considered in interpreting the results of this study. First, the study was conducted at the outpatient rehabilitation clinics of a rehabilitation center and a university medical center, both in the Netherlands. There is a need to explore whether the tool is applicable in other health settings, especially other health care systems. Second, we involved adult clients with a first-time stroke or multiple sclerosis. We intended to involve clients with cerebral palsy, but none of them had a health problem requiring multidisciplinary rehabilitation. There is a need to study whether the tool is also applicable in clients with other diagnoses, although we expect the tool to be broadly applicable. Third, not all clinicians could participate in the focus groups, due to scheduling issues. To overcome this, we interviewed clinicians individually, and we asked clinicians to also report the experiences of other clinicians with the same disciplinary background. Fourth, in this formative evaluation, we used a qualitative method and we relied fully on self-report. Furthermore, the chaplain-researchers participated in the session on global meaning and they performed the evaluation interviews. It is, of course, preferable that the evaluation is independent of the intervention. A quantitative evaluation is needed of the impact of the tool on meaningfulness of rehabilitation goals, motivation, behavior change, and health outcomes, using self-reported as well as observed measures, in a study with high methodological quality.

The above considerations result in a number of suggestions on the further development, implementation, and evaluation of the tool. It is suggested (i) to determine for which clients this tool has added value, and at what stage of rehabilitation; (ii) to improve the translation of improved rehabilitation motivation into behavior change; (iii) to consider who participates in the session on global meaning; (iv) to involve all members of the team in the implementation of the tool, from the very beginning; (v) to determine the effect of using the tool on the meaningfulness of rehabilitation goals, motivation, behavior change, and health outcomes, in quantitative studies.

In conclusion, clients as well as clinicians experienced the tool as helpful in setting meaningful goals in rehabilitation. The tool helped to set a meaningful overall rehabilitation goal and specific goals that became meaningful as they serve to achieve the overall goal. This contributed to clients’ rehabilitation motivation. In some clients, the meaningfulness of the rehabilitation goals facilitated the process of behavior change. The formative evaluation yielded valuable suggestions for the further development, implementation and evaluation of the tool.

### Clinical messages

- Clients and clinicians both reported that our tool helped to set a meaningful overall rehabilitation goal and specific goals that became meaningful as they served to achieve the overall goal.
- Because the rehabilitation goals were experienced as meaningful, clients’ intrinsic rehabilitation motivation increased.
- The meaningfulness of the rehabilitation goals facilitated the process of behaviour change to a limited extent.

## Supplemental Material

sj-pdf-1-cre-10.1177_02692155211046463 - Supplemental material for Setting meaningful goals in rehabilitation: A qualitative study on the experiences of clients and clinicians in working with a practical tool Click here for additional data file. Supplemental material, sj-pdf-1-cre-10.1177_02692155211046463 for Setting meaningful goals in rehabilitation: A qualitative study on the experiences of clients and clinicians in working with a practical tool by Elsbeth Littooij, Suzan Doodeman, Jasmijn Holla, Maaike Ouwerkerk, Lenneke Post, Ton Satink, Anne Marie ter Steeg, Judith Vloothuis, Joost Dekker and Vincent de Groot in Clinical Rehabilitation
