# Supplementary material for: Setting meaningful goals in rehabilitation: A qualitative study on the experiences of clients and clinicians in working with a practical tool
Source: Clin Rehabil. 2021 Nov 3;36(3):415–28. doi: 10.1177/02692155211046463 (PMC8850761; doi:10.1177/02692155211046463)
Supplement: sj-pdf-1-cre-10.1177_02692155211046463 - Supplemental material for Setting meaningful goals in rehabilitation: A qualitative study on the experiences of clients and clinicians in working with a practical tool [file sj-pdf-1-cre-10.1177_02692155211046463.pdf]

Supplementary file 1

Topic list for interview with clients.

During your rehabilitation trajectory, you participated in the project on meaningful goals. Hence this conversation.

1. Can you briefly tell us something about your rehabilitation process.
2. What did you want to achieve?
3. How did you experience the conversation you had with the doctor and the chaplain?
4. Do you remember what overall goals were agreed upon?
5. Has this changed over time?
6. Was (were) your overall goal(s) clear in the initial conversation or did it only become clear over time?
7. What were you working on in the various therapies? How was that determined? (linked to global goal?)
8. Was your goal achieved?
9. What helped you achieve that goal? Certain therapy/therapist, something completely different.
10. Were you motivated to achieve your goal, why?

Topic list for focus groups with physicians.

You have worked with clients who are participating in the project on meaningful goals. Can you recall them?

1. Can you tell each other what it was like to participate in the project?
2. How do you like doing this?
3. How did you do with exploring global meaning?
4. How did it go with articulating the overall goals, easy/difficult?
5. How was the collaboration with the chaplain?

6. How did global goals affect the rehabilitation process? For the client, and for you as a physician? What did you hear back from other members of the team? If necessary, explicitly: did you notice influence on client motivation? Did the goals change?

7. What does this mean for your own way of working?

8. And for the team?

9. Is the session on meaningful goals held at the right time in the rehabilitation process?

10. Is the session done with the right people (physician and chaplain)?

11. Do you see this session about global meaning and goals as adding to the rehabilitation process?

<u>Topic list for focus groups with other members of the rehabilitation team</u>

You have worked with clients who are participating in the project on meaningful goals. Can you recall them?

1. Can you tell us about working with global meaning and the overall goals?

2. Did this intervention have an impact on rehabilitation (e.g., did people feel more engaged, was there more motivation, etc.)?

3. Did any of the clients experience changes in global goals or specific goals along the rehabilitation trajectory? (Was there a need for that, did people indicate that, etc.)

4. What does this mean for your own way of working?

5. And for that of the team?

6. Do you see this session about global meaning and global goals as adding to the rehabilitation process?

7. Is the session on meaningful goals held at the right time in the rehabilitation process?

8. Is the session done with the right people (physician and chaplain)?

Supplementary file 2

Development of themes

Initially three groups of themes were formulated, all with a sub-theme: (1) Motivation - with as sub-theme 'global meaning as source of behavior change', (2) 'stability of global meaning and goals' – with 'discrepancy' as a sub-theme, and (3) 'process' – with the sub-theme 'global meaning as core business'.

After discussing the themes and their arrangement with the research group, we changed the names of the first themes in 'impact on motivation' and 'impact on behavior change'. The theme of 'stability in global meaning and goals' remained the same, whereas 'discrepancy' was changed in 'existential distress'. The theme 'process' was changed into 'process of setting meaningful goals'. Besides that, the theme 'overall evaluation' was created, which comprised the overall appreciation of the tool, as well as the sub-theme 'global meaning as core business'.

Motivation

One of the core points of attention regarding the involvement of global meaning in rehabilitation was the effect on clients' motivation. In the analysis of interviews and focus groups, motivation was used as an initial code. In a later stage of analysis, it was also used as a theme, comprising codes such as 'motivation', 'relationship patient-professional', 'system vs patients' needs', 'heavy', 'what moves a person', 'get to work'.

Global meaning as source of behavior change

Related to motivation, behavior change occurred as an important theme for clients as well as professionals. Codes that were grouped under this theme were 'looking differently', 'enjoying consciously', 'struggle', 'change of mindset', 'sustainability of behavior change', 'focusing on adapted goal'.

Stability of global meaning and goals

Initially we created the themes ‘change in global meaning and goals’ and ‘stability of global meaning and goals’, which we later combined. Under this combined theme we gathered codes such as ‘continuity’, ‘change in values’, ‘trust’, ‘insight gained during rehabilitation’, ‘adaptation’. These codes referred to stability or to changes that were experienced as non-problematic, or logical, or even desired changes. When reviewing these codes we took into account the specific rehabilitation goals we extracted from the clients’ files.

Existential distress In some cases changes appeared to be problematic. Codes such as ‘ambivalence’, ‘existential problem?’, ‘growing awareness’, ‘change of identity’, ‘values interfere with acceptance’ lead us to create the theme ‘existential distress’.

Process of setting meaningful goals

In the same way as motivation, process was used as an initial code, and later on also as a theme. It comprised codes such as ‘preparation’, ‘timing’, ‘active use of the tool’, ‘different conversation?’, ‘tool as thermometer’, ‘context’, ‘evaluation’, ‘making choices’, ‘professional view’, ‘by whom’, ‘which patients?’.

Overall evaluation

Initially we focused on goalsetting, using global meaning as an instrument to set meaningful goals. In the interview phase, not so much goals, but global meaning as such was appreciated by clients as well as professionals in fostering the rehabilitation process. In one of the focus groups, a professional stated that global meaning is core business of rehabilitation. Under this theme codes such as ‘appreciation of tool’, ‘global meaning more important than goals’, ‘relation global meaning and (global) goals’ were grouped. In a later stage of the analysis, this theme was merged with the theme ‘overall evaluation’, which comprised codes such as ‘attuning to’, ‘wanting more’, ‘effect’, ‘felt good’, ‘good enough’.
